# Implementation of the goal-setting components in popular physical activity apps: Review and content analysis

**DOI:** 10.1177/2055207619862706

**Published:** 2019-07-16

**Authors:** Dario Baretta, Paulina Bondaronek, Artur Direito, Patrizia Steca

**Affiliations:** 1 Department of Psychology, University of Milano-Bicocca, Italy; 2 eHealth Research Unit, University College London, UK; 3 Yong Loo Lin School of Medicine, National University of Singapore, Singapore; 4 Centre for Behaviour Change, University College London, UK

**Keywords:** Content analysis, digital health, goal-setting components, goal setting theory, mHealth, mobile apps, physical activity, review

## Abstract

**Objective:**

Goal setting is an effective strategy to promote physical activity. Commercial apps that tackle physical activity often include goal setting; however, it is unknown whether the implementation of the goal-setting components is congruent with the theory. This study evaluated the quality of goal setting in popular free and paid physical activity apps by assessing the presence of effective goal-setting components.

**Methods:**

A six-item scale was developed based on the goal-setting literature and used for coding each app for the presence/absence of goal-setting components (i.e. specificity, difficulty, action planning, timeframe, goal evaluation and goal re-evaluation). Cohen’s Kappa was used to evaluate inter-rater reliability for each scale item. The number of goal-setting components included in the 40 apps was calculated and the difference between free and paid apps was assessed.

**Results:**

All scale items achieved satisfactory inter-rater reliability except ‘goal evaluation’. The most frequently included goal-setting components in popular physical activity apps were ‘goal specificity’ (95% of the apps) and ‘goal timeframe’ (67.5%). Conversely, only 47.5% and 25% of the apps implemented ‘action planning’ and ‘goal difficulty’, respectively, and none included ‘goal re-evaluation’. No differences emerged between free and paid apps.

**Conclusions:**

The quality of the goal-setting strategy in popular physical activity apps could be improved by introducing components scarcely implemented to date. In particular, tailoring the goal difficulty to the users’ ability level and re-evaluating the goals based on achievements should be implemented to increase the quality of goal setting.

## Introduction

Mobile applications (apps) represent one of the widespread and accessible *Digital Behaviour Change Interventions*^1^ to promote physical activity (PA). The plethora of health apps available in the major app stores is continuously growing, as is the number of app downloads.^2^ However, one of the major issues associated with existing PA apps concerns their effectiveness in promoting PA.

PA apps available in the app stores are rarely evidence based or evaluated using high standard research methods, such as randomised controlled trials.^3–6^ In addition, randomised controlled trials can be slow and resource-heavy,^7^ hence they may not be feasible considering the scale of the health app market. In the absence of high-quality evidence of effectiveness, previous studies attempted to assess the quality of the apps by focusing on adherence to theory, most commonly the presence of behaviour change theory, operationalised as the inclusion of behaviour change techniques (BCTs).^8^ Results evidenced that the most common BCTs included in PA apps were self-regulatory BCTs, such as goal setting, self-monitoring, and feedback on behaviour.^9–12^ Self-regulatory BCTs have been shown to be associated with effectiveness of interventions targeting PA.^13,14^ Among this group of BCTs, the role played by goal setting is particularly crucial in promoting PA behaviour change.^15,16^ While feedback on behaviour and self-monitoring provide objective behavioural parameters, goal setting implies a prospective and proactive decision about the future standard (i.e. target behaviour) at the basis of the self-regulatory process. The relevance of goal setting in behaviour change has been acknowledged by prominent theories, as highlighted by Michie and colleagues,^17^ and supported by meta-analytic evidence.^18^

Even though previous reviews showed that a goal-setting BCT is often implemented in PA apps,^8^ the quality with which such a BCT was delivered remains unclear. While assessing the presence of BCTs allows us to specify the minimum content of *what* is delivered (i.e. fidelity of delivery), it does not allow us to specify the quality of delivery.^19^ For instance, adaptive behaviour change interventions focusing on goal setting to promote PA^20,21^ enable adjustment of the goal based on variables such as previous achievements (e.g. number of steps/day) and contextual changes (e.g. weather, location, time of the day). Conversely, other goal-setting strategies proposed goals that are fixed and do not change over time. Even though implementing the same BCT, these two approaches are qualitatively different (adaptive versus fixed goal setting), with consequent differences in terms of intervention effectiveness.^20,22^

Goal-setting theory^23–25^ proposed specific goal components that should be addressed when setting behavioural goals. These mainly relate to the specificity, difficulty and timing of the goal being set. For instance, in addition of being specific and measurable, goals should be adequate and tailored to the individual skills (i.e. realistic but challenging goals), and be defined in terms of timeframe. Furthermore, the effect of goal setting on exercise was proposed to be moderated by additional components, namely action planning, goal evaluation and goal re-evaluation. Once a personal goal has been set, the presence of such components allows adjustment of the level and direction of the effort, thus increasing the potential effectiveness of the goal-setting strategy. Goal-setting components have been widely proposed to impact on the likelihood of effectiveness of behaviour change interventions.^25,26^ Furthermore, previous research syntheses evaluating the effectiveness of goal-setting components on PA behaviour change interventions found that multi-component goal-setting interventions are generally effective in promoting PA behaviour.^15,16^ Therefore, goal-setting components can be considered as a theoretical and evidence-based guide for assessing the quality of PA interventions that implement goal-setting strategies.^15,16,25^ To date, however, no studies have assessed the quality of the goal setting in popular PA apps, defined as the presence of goal-setting components.

The aim of this study was to evaluate the quality of goal setting in popular publicly available PA apps by assessing whether and how goal-setting components were implemented in popular PA apps. The specific objectives were to: (1) describe and quantify the presence of evidence-based components of goal setting in PA apps, and (2) assess the presence of goal-setting components in free versus paid apps.

## Methods

### Study design

The present study was a review and content analysis of the implementation of goal-setting components in PA apps.

### App screening process

The sample was identified from 400 highly ranked free and paid apps from the ‘Health & Fitness category’ in iTunes and Google Play stores to represent the apps that were most visible to the user. This method of assessing popularity has been used in other studies assessing apps.^27–29^

The inclusion criteria were:

- Behaviour targeted is PA.
- The app targets adults.
- The app had stand-alone functionality (i.e. does not need to be linked with peripheral devices, such as pedometers; and does not require paid membership to access the app).

Exclusion criteria:

- Primary aim of the app is focused on multiple behaviours or behavioural outcomes (e.g. apps targeting both PA and diet), as it would be difficult to compare apps targeting multiple health behaviours.
- The app focuses on increasing PA as part of management of a long-term condition, as it may not be comparable to apps targeting PA levels in healthy individuals.
- Apps sold in a pack (i.e. ‘bundle’) were excluded, as it is impossible to extract the popularity ranking from a pack of multiple apps. These apps are very often positioned within the ranks separately.

### Sample identification

The target sample for the present study was 40 PA apps. The sample was obtained from a previous review and content analysis of the most popular PA apps (for details see Michie et al.^8^), which randomly selected and assessed 65 apps for the presence of BCTs. This previous review investigated top-ranked free and paid apps from iTunes and Google Play. Identifying the sample of apps for the current study from the previous review ensured time efficiency and that the most popular PA apps were assessed. As the apps were similar in terms of their primary function, it was deemed that 40 apps would suffice to characterise the popular apps from the market. Five app types/categories were identified in terms of their primary function: workouts (*n* = 31 apps), tracking of movement (*n* = 13), running programmes (*n* = 12), step counter apps (*n* = 6) and interval timers (*n* = 3).

The summary of the app sample with app types/categories in presented in Table 1. Apps that met the inclusion criteria were identified until a total *N* = 40 was achieved. As interval timers did not implement goal-setting strategies, this app type was excluded from the present study. During screening, only one paid step tracker app was retrieved, hence the first available tracking of movement app was added to the sample as it was the next app from the sample of top-ranked apps (i.e. most popular app).

**Table 1.** The summary of the app sample with app types/categories.

App type	Free	Paid
Workouts	10	10
Tracking of movement	5	4
Running programmes	4	4
Step counter	2	1
Total	21	19

### Measures

Goal setting theory^24,25^ and its application to exercise and sport contexts^30,31^ were used as theoretical references for (i) identifying the components of appropriate goal-setting strategies to support PA, and (ii) specifying the evaluation criteria for assessing the quality of goal setting in PA apps. Furthermore, recent reviews of goal-setting strategies in behaviour change interventions targeting PA, diet, and smoking cessation served as references for refining the evaluation criteria^32^ and defining the scoring of the resulting scale.^33^

The following components identified in the literature were used in the current study to assess the quality of goal setting in PA apps:

1. Specificity: Specific goals have been shown to lead to higher levels of performance than no goals or general ‘do your best’ goals. Goals should be measurable and defined in behavioural terms.^25,30^
2. Difficulty: even though goal difficulty is associated with performance, goals should be commensurate to the individuals’ ability level to ensure that goal achievement constitutes a challenging but doable task at the same time.^25^ If goals are too difficult they can lead to failure and undermine self-efficacy beliefs, while goals that are too easy can lead to mediocre performance or demotivation.^26^ For these reasons, an early assessment of the individual skills is required to define the most appropriate goals.
3. Timing/timeframe: Research in sport contexts has constantly evidenced the importance of setting both short- and long-term goals.^31^ Long-term goals provide the direction and final destination for individuals, while short-term goals are important to provide feedback on progress towards the long-term goal. When both long- and short-term goals are not present, goal-setting research in exercise^16^ and primary care^34^ has shown that interventions targeting short goals alone are more effective than interventions that target long-term goals exclusively. However, the definition of the goal-setting timeframe in exercise and PA promotion is somewhat vague. In a sport context, long-term goals mainly refer to seasonal or Olympic goals, whereas in interventions targeting PA the length of interventions is extremely heterogeneous, varying from a couple of weeks to 1 year.^16^ In the exercise and PA promotion context, there are no clear and well-defined time boundaries that differentiate short-term from long-term goals. Based on the timeframe of goal setting in current PA apps, for the purpose of the present study we considered daily goals as short-term, and weekly or farther goals as long-term goals.
4. Action planning: Goals should be supported by a plan that specifies the modalities in which the goal will be achieved. By specifying when, where, and how to act, action planning helps individuals to implement their intentions and initiate the goal faster than those who do not form action plans.^35^ For this reason, it is important to make specific plans that describe how the goal will be achieved.
5. Goal evaluation: Performance should be constantly evaluated, highlighting individual progress towards the goal. The evaluation process allows individuals to adjust their behaviour (i.e. effort and strategies) to match what the goal requires or to redefine goals based on failures or successes.^25^
6. Goal re-evaluation: Based on goal evaluation outputs, goals should be constantly redefined in order to set new ones that are commensurate to individuals’ achievements and progresses. When individuals constantly exceed their set goals or, conversely, repeatedly do not reach them, it might be the case to review behaviour goals (i.e. redefine more difficult or easier goals).^25,30^ The goal evaluation and goal re-evaluation constitute an iterative loop that helps individuals to progress over time and sustain their motivation and commitment by providing achievement experiences and preventing failure.

Based on these components, a scale was developed to assess the degree to which such components are implemented in PA apps (see Table 2).

**Table 2.** Evaluation criteria and scoring for each goal-setting component.

Component	Evaluation criteria	Answer (score)
Specificity	Is the goal set in specific and measurable behavioural terms?	No (0)Yes (+1)
Difficulty	Is the goal set considering the users’ skill or expertise level?	No (0) Yes (+1)
Timing	Is the goal set by specifying the timeframe? If yes, which is the timeframe?	No (0) Yes a. daily (+1) b. weekly or more (+.5) c. both (+1.5)
Action planning	Does the app prompt the users to develop or facilitates the development of action plans as a strategy to achieve goals?	No (0) Yes (+1)
Goal evaluation	Does the app provide evaluative feedback about the users’ progress towards the goal?	No (0) Yes (+1)
Goal re-evaluation	Does the app adjust and redefine goals on the basis of users’ achievements and failures?	No (0) Yes (+1)

### Procedures

Two researchers (DB, PB) downloaded the 40 apps identified and familiarised themselves with the app functionalities, focusing on features related to goal setting. Ten apps were user-tested and independently assessed for the presence/absence of each goal-setting component, after which the researchers met to compare results and discuss discrepancies. Consequently, the assessment tool was refined, and the remaining 30 apps downloaded and independently evaluated. Disagreements were resolved through discussion between DB and PB following the completion of app coding.

### Data analysis

Inter-rater reliability was assessed on all the 40 apps using Cohen’s Kappa with two raters.^36^ Cohen’s Kappa cut-off values considered for the study are described in McHugh.^37^ For the purpose of the present study we decided to consider a less restrictive cut-off value of .50 and solving potential disagreement at a later stage. We anticipated coding discrepancies due to the novelty of the coding instrument and because the assessment of the implementation of goal-setting components was inferred from app user-interfaces rather than from text descriptions. A score was assigned to each specific app on the basis of the agreed components. Quantitative data were analysed using frequencies and percentages of the scores of each goal-setting component. Differences in terms of overall quality of goal setting between free and paid PA apps were tested with *t*-test statistics.

## Results

Results indicated levels of agreement varying from none to almost perfect (see Table 3). The item goal evaluation was removed from the scale since the level of agreement between raters was below .50.

**Table 3.** Cohen’s K for the items representing the quality of goal setting in physical activity.

	Cohen’s Kappa	Confidence intervals	Level of agreement
Specificity	.53*	(.06, 1)	Weak
Difficulty	.73**	(.49, .97)	Moderate
Timing	.72**	(.54, .90)	Moderate
Action planning	.64**	(.40, .88)	Moderate
Goal evaluation	.05	(−.11, .21)	None
Goal re-evaluation	1**	–	Almost perfect

*p<.01; **p<.001

As shown in Table 4, 95% of the apps (38/40) set goals that were specific and measurable while just 10 apps out of 40 (25%) tailored the difficulty of the goal to the user level of expertise. Timing is one of the most common goal-setting components in PA apps: 27 apps (67.5%) specify or ask the user to specify the timeframe for achieving the goal. Specifically, 23 apps (57.5%) included a goal timeframe that comprehends both proximal and distal goals, one app (2.5%) adopted a proximal timeframe alone, and three apps (7.5%) included a distal timeframe alone. Furthermore, 47.5% of the apps (19/40) provided a strategy to enable users to make specific plans to achieve the goal. The goal re-evaluation component was absent in the sample of PA apps assessed.

**Table 4.** Frequencies of the presence of each goal-setting component in PA apps.

Goal-setting component	Free (*n*=21)	Paid (*n*=19)	Total (*n*=40)	
Specificity	20	18	38	(95%)
Difficulty	5	5	10	(25%)
Timing	14	13	27	(67.5%)
Proximal	1	–	1	(2.5%)
Distal	3	–	3	(7.5%)
Proximal and distal	10	13	23	(57.5%)
Action planning	9	10	19	(47.5%)
Goal re-evaluation	–	–	–	(0%)

Presence of goal-setting components and score for each app are shown in Table 5. The *t*-test statistics indicated that there were no mean differences between free and paid apps with respect to score of goals setting components (t(38) = –.77, *p* = .45), suggesting that they did not differ with respect to quality of implementation of the goal-setting strategy.

**Table 5.** Goal-setting components and overall score for each of the apps and mean score for free and paid apps (scale 0–5.5 points).

Name of the app	Price	Type	Goal-setting component	Score
30 Day Ab Challenge FREE	Free	W	S, D, Tpd, AP	4.5
Sworkit – Custom Workouts for Exercise & Fitness	Free	W	S, D, Tpd, AP	4.5
7 Minute Workout by Simple Design Ltd	Free	W	S, D, PD	3.5
Fitness & Bodybuilding	Free	W	S, Tpd, AP	3.5
Home workout MMA Spartan Free	Free	W	S, Tpd, AP	3.5
5K Run - Couch to 5K	Free	RP	S, Tpd, AP	3.5
C25K® - 5K Running Trainer	Free	RP	S, Tpd, AP	3.5
Couch to 10K Running Trainer	Free	RP	S, Tpd, AP	3.5
One You Couch to 5K	Free	RP	S, Tpd, AP	3.5
Fitbit	Free	T	S, PD	2.5
Daily Workouts FREE	Free	W	S, AP	2.0
FitNotes - Gym Workout Log	Free	W	S, D	2.0
Freeletics Bodyweight – Workout and Training	Free	W	S, D	2.0
Steps Pedometer & Step Counter Activity Tracker	Free	S	S, Tp	2.0
Map My Run – GPS Running & Workout Tracker	Free	T	S, Td	1.5
Pacer – Pedometer plus Weight Loss and BMI	Free	S	S, Td	1.5
Running, Walking and Biking with Endomondo	Free	T	S, Td	1.5
7 Minutes Workout – Women Fitness Exercise Trainer	Free	W	S	1.0
Runtastic Results: Body Workout Fitness Trainer	Free	W	S	1.0
Runtastic Running & Fitness	Free	T	S	1.0
Strava	Free	T	-	0.0
Mean				2.45
WalkJogRun GPS Running Routes	Paid	T	S, D, Tpd, AP	4.5
7 Minute Workout Pro	Paid	W	S, D, Tpd	3.5
Adrian James 6 Pack Abs Workout	Paid	W	S, D, Tpd	3.5
Adrian James High Intensity Interval Training	Paid	W	S, D, Tpd	3.5
Fitness Trainer FULL version	Paid	W	S, Tpd, AP	3.5
Full Fitness : Exercise Workout Trainer	Paid	W	S, Tpd, AP	3.5
MapMyFitness+ Workout Trainer	Paid	W	S, Tpd, AP	3.5
Push ups 0 to 100	Paid	W	S, Tpd, AP	3.5
Map My Walk+ GPS Pedometer	Paid	T	S, Tpd, AP	3.5
10K Running Trainer Pro	Paid	RP	S, Tpd, AP	3.5
5K to 10K	Paid	RP	S, Tpd, AP	3.5
Couch to 5K Runner, 0 to 5K run training	Paid	RP	S, Tpd, AP	3.5
Marathon Trainer - 26.2 42K	Paid	RP	S, Tpd, AP	3.5
iMuscle 2 – iPhone Edition	Paid	W	S, D	2.0
Instant Fitness : 600+ exercises, 100+ workouts…	Paid	W	S	1.0
Police Fitness – Bleep Test	Paid	W	S	1.0
Runtastic Mountain Bike PRO GPS Biking Computer, Trail and Route Tracker	Paid	T	S	1.0
Runtastic Road Bike PRO	Paid	T	S	1.0
Footsteps – Pedometer	Paid	S	-	0.0
Mean				2.76
Overall mean				2.61

Note: all apps are grouped by price and ranked by goal-setting score;

Type abbreviations = W: workout app; S: step counter; T :tracking of movement; RP: running programmes;

Goal-setting component abbreviations = S: specificity; D: difficulty; Tpd: proximal and distal timing; Tp: proximal timing; Td: distal timing; AP: action planning.

## Discussion

### Principal findings

This study evaluated the quality of goal setting in publicly available popular PA apps by assessing whether goal-setting components were implemented in those PA apps. Overall, popular PA apps lack theory-based goal-setting components that are key for the efficacy of the goal-setting strategy. Specifically, results showed that 38 apps (95%) set specific and measurable goals, allowing each user to evaluate whether the goal has been achieved or not. However, the implementation of the remaining goal-setting components was limited, and goal re-evaluation was absent from app features. There were no differences in the score of goal-setting components between free and paid apps.

Remarkably, only 10/40 apps (25%) tailored the goal difficulty to the users’ experience and ability level, highlighting opportunities to enhance the behaviour change potential of PA apps. Individuals are motivated and committed with their goal if the goal is achievable but challenging at the same time,^26,38^ and it is known that tailoring intervention content positively influences engagement with digital interventions.^39^

Nearly 70% of the apps (*n* = 27) proposed or allowed users to set a scheduled goal. Goal timeframes help users to better specify the goal and distribute effort over time. However, a combination of short- and long-term goals has been shown to be most effective in setting PA goals in exercise^16^ and sport.^31^ Among apps including goal timeframes, 23 out of 27 implemented both daily and weekly goal options, supporting users according to scientific recommendations.

Furthermore, 47.5% of the apps (19/40) provided features that enabled action planning. This is similar to previous studies that assessed the presence of BCTs in PA apps.^9,12^ It is important that specific plans are set to facilitate the effectiveness of goal-setting strategies and, consequently, to promote goal attainment.^35^ In agreement, recent qualitative research found that tailored action planning features are deemed important by users for promoting engagement with commercial PA apps.^40^ Therefore, commercial PA apps could benefit from the implementation of action planning features, for instance by acknowledging potential facilitators and barriers of goal achievement (e.g. weather, weekdays/weekends).^41^

Behaviour change strategies are not effective under all conditions, and their translation onto usable intervention elements, such as app features, should consider their parameters for effectiveness.^42^ This study pinpointed clear opportunities for improvement of the goal-setting strategy in commercial PA apps. Previous findings showed significant positive effects on PA when goal modifications occur on the basis of individual achievements,^16^ probably because individuals, as suggested by Bandura,^38^ are more likely to be confident and stay motivated if goals are re-evaluated and adjusted as a function of achievement. Goal setting is an iterative process of continuous cycles of evaluation and goal re-definition, rather than a static decisional point.^30^ In support, digital interventions assigning PA goals that adapt to individual achievements (e.g. level of PA over time) were more effective in promoting PA than interventions where goals were static.^20^

Adapting app content to the user’s progress has also been hypothesised as an effective strategy to improve engagement with digital interventions targeting health-related behaviours.^43^ However, in the current study, app features implementing goal setting were largely fixed and unresponsive to changes in the user’s performance. Specifically, none of the apps provided the opportunity to re-evaluate the goal, even though digital technology enabling goal re-evaluation based on achievement has been developed for some time.^44^ Recent advanced methodologies in the field of digital interventions, such as Just in Time Adaptive Interventions,^45^ seem particularly suited for developing PA apps that leverage time-varying information (e.g. the user’s progress towards the goal) to implement dynamic goal-setting strategies.

### Strengths

This study followed a systematic method of sample identification and assessment. First, the sample was identified through screening of the 400 most popular apps, likely representative of apps that many individuals have downloaded and used. Second, the sample was assessed independently by two reviewers by user-testing the apps, rather than basing the app evaluation on text descriptions found in the app stores.

### Limitations

Although apps were left running on the smartphones to assess any features related to goal setting that were not evident at all times, it is possible that some of the features were missed. Second, even though a Kappa below .60 is suggested to indicate inadequate agreement,^37^ we used a less restrictive cut-off value of .50 as we envisaged some discrepancies due to the novelty of the assessment tool and because of the implementation of goal-setting component was inferred from graphical details in the apps rather than from a scientific description of behaviour change interventions. However, this is the first attempt to characterise goal-setting components in popular apps; discrepancies between raters were discussed, and the instrument was revised and improved accordingly. Third, the ‘goal evaluation’ goal-setting component did not achieve satisfactory agreement between raters and was excluded from the scoring form. As implemented in current PA apps, ‘goal evaluation’ design prevented a clear distinction from ‘feedback on behaviour’. The distinctive characteristics of these two components were based on small graphical differences, which were not interpreted similarly by raters. Finally, the generalisability of our findings warrant caution as it is possible that some features of goal-setting components in current PA apps were not captured in our study sample. However, we user-tested the most popular and commonly downloaded PA apps in iTunes and Google Play, which ensures we assessed a sample of apps that many individuals are using, and this therefore increases the relevance of our study.

### Implications

First, research is needed to assess the quality of delivery of theory-based constructs through app features. Second, there is a need to understand how users utilise the PA apps and how particular components of the goal setting can be adapted to the user to facilitate PA behaviour.

Developers of digital interventions should consider the inclusion of the full range of components of the goal setting to increase the behaviour change potential of the PA apps. Tailoring the goals to user’s needs and re-evaluating the goals based on the performance should be utilised in the PA apps. The assessment criteria used in this study can be used as a guidance to develop the goal-setting features of apps.

## Conclusions

This study showed that the quality of the goal-setting strategy in PA apps could be improved. Some of the components comprising the most effective goal-setting strategy to increase PA have been utilised infrequently, or not utilised at all, in publicly available popular PA apps. Apps enable convenient features that facilitate monitoring of goal attainment, such as automatic record of PA sessions and convenient logging of behaviour. However, there is a vast opportunity for improvement of goal-setting features, specifically by tailoring the goal setting to the user and adapting it according to user performance to increase the likely efficacy of PA apps.
